# Deep coma in a child treated with propranolol for infantile hemangioma

**DOI:** 10.1186/s12887-019-1598-0

**Published:** 2019-07-02

**Authors:** Ilirjana Bakalli, Elmira Kola, Robert Lluka, Ermela Celaj, Durim Sala, Inva Gjeta, Sashenka Sallabanda, Dea Klironomi

**Affiliations:** 1 Pediatric Intensive Care Unit, University Hospital Center “Mother Theresa”, Rruga e Dibres, 376 Tirana, Albania; 2 Medical University of Vienna, Vienna, Austria

**Keywords:** Infantile hemangioma, propranolol, adverse effects

## Abstract

**Background:**

Propranolol hydrochloride is the first-line agent recommended for the treatment of infantile hemangiomas (IH). Serious adverse effects of propranolol therapy for hemangiomas are infrequent.

**Case presentation:**

We report a case presented in deep hypoglycemic coma during his treatment with propranolol for IH. Through our case report and the review of the literature, we aimed to underline the importance of recognizing adverse effects during propranolol therapy. Although propranolol has a long history of safe and effective use in infants and children, pediatricians should be aware that life-threatening adverse effects can happen during propranolol therapy for IH.

**Conclusion:**

Early identification of these adverse effects can be of great importance for patient management and prognosis. It must certainly be noted that not just early identification among doctors, but education for parents is crucial.

## Background

Infantile hemangiomas (IH) are benign tumors of vascular endothelium affecting about 5-10% of infants [1–9]. The approach to the treatment of IH should be individualized, based upon the size of the lesion, morphology, location, presence or possibility of complications [10, 11]. Oral propranolol is the first line agent recommended for the treatment of IH. Serious adverse effects of propranolol therapy for hemangiomas, which include hypotension, bradycardia, hyperkalemia, bronchospasm and hypoglycemia, are infrequent [7, 12–16]. We describe one case receiving treatment with propranolol hydrochloride for his IH, who presented in deep coma. We aim to highlight the importance of recognizing adverse effects during propranolol therapy for both physicians and caregivers.

## Case presentation

A healthy 13 month-old boy was receiving oral propranolol hydrochloride for a large IH. There was no history of hypoglycemia or other medications. The initial dose of propranolol hydrochloride was 0,5 mg/kg/day and over several weeks was titrated to 1mg/kg/d. He received the treatment for about 9 months and had a significant reduction in the size of IH. The night prior to hospitalization, the child looked tired, drowsy and had a very poor oral intake. Due to his sleepiness, he didn’t receive the evening dose of propranolol. With the child's poor feeding, it was important to skip the propranolol dose and hold it temporarily. Since his mother had no clear information about the importance of taking propranolol with food, as well as when to stop temporarily the propranolol, she gave him the missed dose during the night, without food (about six hours before hospital admission). In the morning the child was unresponsive, unarousable, with a grey pallor. The clinical situation was critical: in a deep coma, with a severe hypothermia (34°C), cold sweats, slow heart beats (60-65 beats/minute) and a low blood pressure (85/46 mmHg). After immediate suspicion, we confirmed a severe hypoglycemia (26 mg/dl). After confirming the hypoglycemia, we asked about the presence of any medication at home, in order to exclude the possibility of a drug poisoning. The mother didn’t inform us at the first moment that the child was taking propranolol for IH. No advices were given to the parents about adverse effects of propranolol and how recognizing signs of its serious adverse effects (including hypotension, bradycardia, wheezing, and hypoglycemia). The child was given initially 30 ml of 10% dextrose solution intravenously, which resulted in stabilization of his glucose level. We continued the treatment with 7.5% dextrose for a few hours after. Clinical condition was completely normalized.

## Discussion

IH grow rapidly in the first 3 months after birth and usually stop growing between 6 and 12 months of age. Most IH cause no problems and do not require treatment, however, they need to be treated if they develop in a position that interferes with a vital function such as breathing, feeding or vision [1–5, 17]. .It is reasonable to stop the therapy after the proliferation, but in our country we continue treatment up to 12 to 18 months of age, based on clinical response. The approach to the treatment should be individualized, by carefully weighing the risk of treatment and the potential benefits [1]. Since 2008, propranolol has been found to be a safe and effective treatment for IH [17, 18]. Over the last decade, numerous cases of successful treatment of IH with propranolol have been reported [1–5, 10, 12]. With almost 1000 hits in the medline for “propranolol and hemangioma”, we found only one randomized, controlled trial of oral propranolol in IH [7, 12]. As more physicians have begun using propranolol as “a miracle drug” for IH, reports of side effects have also increased [6, 7, 9, 14, 15, 19–26].

Serious adverse effects of propranolol therapy for IH, such as hypotension, bradycardia, hyperkalemia, bronchospasm and hypoglycemia are infrequent, but without a known incidence [1, 7, 12–16]. In a review of 906 French children treated with propranolol, one or more adverse effects occurred in 9% of cases [26]. Prashanth G. reported as many as 35 adverse effects among 28 infants, undergoing propranolol therapy for IH, which is considerably high [20]. In opposite to these studies a Chinese study including 1260 children, reported a very low incidence of side effects. In that study the intolerable side effects (e.g. symptomatic hypoglycemia, severe respiratory disorders) was 2.1% (26 patients) [6].

Of the potential serious adverse effects, hypoglycemia is the most worrisome. At our knowledge, this is the first presentation of deep coma during propranolol therapy for IH. Hypotension and bradycardia, along with the severe hypoglycemia has precipitate in a very critical clinical situation for our child. Hypoglycemic coma represents a life-threatening emergency that require prompt intervention for preservation of life and brain function.

Hypoglycemia as a side effect of propranolol therapy needs to be taken seriously, because it is known that repetitive and sustained hypoglycemia in infants may lead to permanent impairment of brain growth and function [20]. Lawley et al. described the first report of hypoglycemia in 2009, only one year after the beginning of propranolol use for IH [13].

Other cases with symptomatic hypoglycemia had been reported in infants with IH who have been treated with oral propranolol [12–14, 21, 23–25]. These cases occurred in both newborns and toddlers, but were often associated with poor oral intake or concomitant infection, as it is found in our case [19]. Although most of the reported patients who developed hypoglycemia were prescribed higher doses of propranolol, there were patients taking relatively small doses [21]. Consistent with previous reports, to our patient was prescribed a relatively low dose of propranolol (1 mg/kg/day), further supporting the concept, that hypoglycemia associated with propranolol therapy may not be dose dependent.

The early clinical signs of hypoglycemia (sweating, jitteriness, irritability, cyanosis, poor feeding, hypothermia, lethargy) may be masked by beta-blockers drugs (Propranolol), except sweating [1, 17]. Thus sweating may be the most reliable sign of hypoglycemia in such cases. To reduce the risk of hypoglycemia, propranolol should be administered during the daytime, with food (shortly before or after administration) [1, 16, 17, 19]. To underline is the fact that propranolol should be discontinued during periods of illness or poor oral intake [5, 17, 19, 21]. While recognition of signs and symptoms of hypoglycemia may prompt early intervention, measures may be taken to decrease the risk of hypoglycemia [19]. In small children, along with limited glycogen stores, we have their inability to communicate symptoms. That’s why detailed education regarding proper administration of the medication as well as warning signs of serious adverse effects are necessary for parents and caretakers and should not be underestimated as in our case.

## Conclusion

Although propranolol has a long history of safe and effective use in infants and children, pediatrician should be aware that life-threatening adverse effects can happen during propranolol therapy for IH. Early identification of these adverse effects can be of great importance for patient management and prognosis. It must certainly be noted that not just early identification among doctors, but education for parents is crucial.

## Data Availability

There are no more specific data that could be shared.

## Abbreviations

IH: Infantile hemangiomas
PICU: Pediatric Intensive Care Unit

## References

[1] Metry DW. Infantile hemangiomas: Management. UpTodate. Last updated may 16, 2018.

[2] Darrow H, Greene AK, Mancini AJ, Nooper AJ (2015). Diagnosis and management of infantile hemangioma. Pediatrics.

[3] Darrow H, Greene AK, Mancini AJ, Nooper AJ (2015). Diagnosis and management of infantile hemangioma. Pediatrics.

[4] Leaute-Labreze C, Harper JI, Hoeger PH (2017). Infantile hemangioma. Lancet.

[5] Martin K, Bleib F, Chamlin S, Chiu YE, Frieden I, Frommelt PC (2013). Propranolol treatment of infantile hemangiomas: anticipatory guidance for parents and caretakers. Pediatr Dermatol.

[6] Ji Y, Chen S, Wang Q, Xiang B, Xu Z, Zhong L (2018). Intolerable side effects during propranolol therapy for infantile hemangioma: frequency, risk factors and management. Sci Rep.

[7] Fette Andreas (2013). Propranolol in Use for Treatment of Complex Infant Hemangiomas: Literature Review Regarding Current Guidelines for Preassessment and Standards of Care before Initiation of Therapy. The Scientific World Journal.

[8] Treating hemangiomas with propranolol. Available at: https://www.gosh.nhs.uk/medical-information-0/medicines-information/treating-haemangiomas-propranolol Last updated: June 2014.

[9] Tosoni A, Cutrone M, Carbonare MD, Pettenazzo A, Perilongo G, Sartori S (2017). Cardiac arrest in a toddler treated with propranolol for infantile hemangioma: a case report. Ital J Pediatr.

[10] Frieden IJ, Eichenfiled LF, Esterly NB, Geronemus R, Mallory SB (1997). Guidelines of care for hemangiomas of infancy. American Academy of Dermatology Guidelines/Outcomes Comittess. J Am Acad Dermatol.

[11] Frieden IJ (1997). Which hemangiomas to treat- and how?. Arch Dermatol.

[12] Leaute-Labreze C, Hoeger P, Mazereeuw-Hautier j GL, Baselga E, Posiunas G (2015). A randomized, controlled trial of oral propranolol in infantile hemangioma. N Engl J Med.

[13] Lawly LP, Siegfried E, Todd JL (2009). Propranolol treatment for hemangioma of infancy: risks and recommendations. Pediatr Dermatol.

[14] De Graaf M, Breur JM, Raphael MF, Vos M, Breugem CC, Pasmans SG (2011). Adverse effects of propranolol when used in the treatment of hemangiomas: a case series of 28 infants. J Am Acad Dermatol.

[15] Ji Y, Chen S, Xiang B, Yang Y, Qiu L (2017). Safety and tolerance of propranolol in neonates with severe infantile hemangiomas: a prospective study. Sci Rep.

[16] Propranolol, oral tablet. Available at: https://www.healthline.com/health/propranolol-oral-tablet Last updated: Jan 17, 2018.

[17] Propranolol for haemangiomas of infancy. British Association of Dermatologists. Available at: http://www.bad.org.uk/shared/get-file.ashx?id=177&itemtype=document.

[18] Jian D, Chen X, Babajee K, Su J, Hu X, Li J (2014). Adverse effects of propranolol treatment for infantile hemangiomas in China. J Dermatol Treat.

[19] Drolet BA, Frommelt PC, Sl C, Haggstrom A, Bauman NM, Chiu YE (2013). Initiation and use of propranolol for of infantile hemangioma: report of a consensus conference. Pediatrics.

[20] Prashanth GP (2012). How “unsafe” is propranolol when used in the treatment of infantile hemangioma?. JAAD.

[21] Holland KE, Frieden IJ, Frommel PC, Mancini AJ, Wyatt D, Drolet BA (2010). Hypoglycemia in children taking propranolol for the treatment of infantile hemangioma. Arch Dermatol.

[22] Schiestl C, Neuhaus K, Zoller S, Subotic U, Forster-Kueber I, Michels R (2011). Efficacy and safety of propranolol as first-line treatment for infantile hemangiomas. Eur J Pediatr.

[23] Bonifazi E, Acquafredda A, Milano A, Montagna O, Laforgia N (2010). Severe hypoglycemia during successful treatment of diffuse hemangiomatosis with propranolol. Pediatr Dermatol.

[24] Fusilli G, Merico G, Gurrado R, Rosa T, Acquafredda A, Cavallo L (2010). Propranolol for infantile hemangioma and neuroglycopenic seizures. Acta Paediatr.

[25] Leaute-Labreze C, Boccara O, Degrugillier-Chopinet C, Mazereeuw-Hautier J, Prey S, Lebbe G (2016). Safety of oral propranolol for the treatment of infantile hemangioma: a systematic review. Pediatrics.

[26] Prey S, Voisard JJ, Delarue A, Lebbe G, Taïeb A, Leaute-Labreze C (2016). Safety of Propranolol Therapy for Severe Infantile Hemangioma. JAMA..

